# Insights into *Aquilaria* phylogenetics through comparative plastomic resources

**DOI:** 10.48130/forres-0024-0028

**Published:** 2024-09-04

**Authors:** Junhu Kan, Liyun Nie, Zenglu Mi, Xiaojin Liu, Daping Xu, Luke R Tembrock, Zhiqiang Wu, Zhou Hong

**Affiliations:** 1 Research Institute of Tropical Forestry, Chinese Academy of Forestry, Guangzhou 510520, China; 2 Shenzhen Branch, Guangdong Laboratory for Lingnan Modern Agriculture, Key Laboratory of Synthetic Biology, Ministry of Agriculture and Rural Affairs, Agricultural Genomics Institute at Shenzhen, Chinese Academy of Agricultural Sciences, Shenzhen 518120, China; 3 School of Medical, Molecular and Forensic Sciences, Murdoch University, Murdoch, WA 6149, Australia; 4 Department of Agricultural Biology, College of Agricultural Sciences, Colorado State University, Fort Collins, Colorado 80523, United States

**Keywords:** Agarwood, *Aquilaria*, Plastomes, Evolutionary markers, DNA barcoding.

## Abstract

The plastid is an essential organelle for its role in photosynthesis and energy production and its genomic information is always employed as important evolutionary markers to explore the relationship among species. Agarwood (*Aquilaria*), prized for its aromatic blend, finds extensive use in various cultures as incense and perfume. Despite its high economic importance, the phylogenetic status among *Aquilaria* based on plastomes remains ambiguous due to the lack of available plastomic resources. To bridge this knowledge gap, 22 *Aquilaria* plastomes were newly sequenced, similar variation patterns in this genus were determined, including a shared 16 bp extension of the *rps19* gene and seven highly variable regions. The analysis highlighted the highest prevalence of the A/T motif among simple sequence repeats in these plastomes. Further phylogenetic analysis revealed *Aquilaria*'s phylogenetic implications with an expanded dataset. This comprehensive plastomic resource not only enhances our understanding of *Aquilaria* evolution but also presents potential molecular markers for DNA barcoding.

## Introduction

Agarwood, a precious aromatic blend, is produced through natural or induced injury to plants within the *Aquilaria* or *Gyrinops* genus of the Thymelaeaceae family^[1]^. As sap seeps from tree wounds mixed with the white wood, a gradual transformation occurs, leading to the development of a distinctive yellow-brown or black-brown wood–oil mixture, thus giving rise to agarwood^[2]^. Renowned for its extraordinary fragrance, agarwood is widely utilized as both incense and perfume across numerous cultures^[3,4]^. Agarwood from various varieties exhibits distinct medicinal properties, boasting significant pharmacological effects, clinical efficacy, and health benefits^[5−8]^. Its exorbitant value has spurred numerous international trades, with annual trading volumes reaching hundreds of tons and trading scales exceeding millions of dollars^[9−11]^. However, its heightened demand has fueled illegal logging and non-selective felling, exacerbated by the scarcity of naturally occurring agarwood trees in the wild^[12,13]^. At present, Appendix II of the Convention on International Trade in Endangered Species of Wild Fauna and Flora (CITS) lists 23 species of *Aquilaria* plants and eight species of *Gyrinops* plants, distributed across countries such as China, Indonesia, Malaysia, India, the Philippines, Cambodia, Vietnam, Laos, Thailand, Papua New Guinea, and Singapore^[14]^. Currently, the agarwood circulating in the market is primarily sourced from several agarwood plants, notably *A. sinensis, A. malaccensis,* and *A. crassna*^[15]^.

When the morphological differences are evident, utilizing such markers offer advantages in terms of easy recognition and understanding, as well as simplicity and intuitiveness. However, since commercially traded agarwood primarily exists in the form of traditional Chinese patent medicines, simple preparations, and wood, often devoid of key morphological components (especially flowers and fruits), there arises a need for an effective method to distinguish between different agarwood species. Plastids, organelles within plant cells, play important roles in photosynthesis and energy generation^[16−18]^. Typically, the plastome presents a conserved quadripartite circular structure, consisting of two single-copy (SC) regions [large single-copy (LSC) and small single-copy (SSC) regions] and two inverted repeat (IR) regions^[19−22]^. Plastomes serve as effective tools for unveiling phylogenetic relationships among plants^[23−27]^. Despite the rapid advancement of high-throughput sequencing technologies, which has resulted in the release of thousands of plastomes in public databases, the number of plastomes from the *Aquilaria* genus remains limited. This scarcity of plastomic data has impeded progress in understanding the evolutionary status of *Aquilaria*.

Although many previous studies have attempted to determine the phylogenetic status of *Aquilaria*, the lack of comprehensive genetic data has limited their efficacy. For instance, Farah et al.^[28]^ utilized five chloroplast loci (*matK*, *rbcL*, *trnL* intron, *trnL-trnF*, and *psbC-trnS*), along with the internal transcribed spacer (ITS) region, in their analysis of *Aquilaria*. However, discrepancies emerged between the phylogenetic trees constructed from these two datasets. Notably, while *A. agallocha* was identified as the sister species to *A. sinensis* based on chloroplast loci, the ITS data revealed a different scenario, with *A. sinensis* clustering together with *A. yunnanensis*. A recent study^[29]^ further revealed a distinct phylogenetic relationship among *Aquilaria* species, which expanded the analysis by including the plastome of *A. rugosa*, resulting in notable changes to the relationships among *A. sinensis*, *A. yunnanensis*, and *A. agallocha*. Additionally, the phylogenetic status of *A. crassna* appeared ambiguous, as different varieties of this species did not cluster together. Specifically, *A. crassna* (MK779998) exhibited a closer phylogenetic relationship with *A. subintegra* (MN147871) than to *A. crassna* (MN125348) varieties. Given these debates and uncertainties, there is a pressing need for a more comprehensive phylogenetic study to provide novel insights into the evolutionary relationships within the *Aquilaria* genus.

In this study, novel sequencing of 22 complete plastomes of *Aquilaria* was conducted, which were combined with 15 published *Aquilaria* plastomes. Subsequently, this dataset was utilized to conduct comparative genomic and phylogenetic analyses, aiming to achieve three primary objectives: (1) addressing the gap of insufficient plastomic resources within the *Aquilaria* genus and gain deeper insights into its evolutionary patterns; (2) identification of highly variable regions (HVRs) suitable for DNA barcode-based identification; and (3) performing a phylogenetic analysis to infer the relationships among *Aquilaria* species using an expanded dataset.

## Materials and methods

### Material collection, DNA extraction, and sequencing

Fresh leaves from five *A. sinensis* samples were collected from the Xishuangbanna Tropical Botanical Garden of the Chinese Academy of Sciences (21°55'55" N, 101°15' 40" E). Additionally, eight *A. sinensis* samples were collected from Jianfeng, Hainan, China (19°47'48" N, 109°48'37" E), while three samples of *A. sinensis* were collected from the Guangdong Academy of Forestry (23°11'38" N, 113°22'52" E). Moreover, four *A. crassna* and two *A. rugosa* samples were obtained from the Agricultural Genomics Institute, Chinese Academy of Agricultural Sciences (22°32'44" N, 114°03'10" E). Genomic DNA extraction from plant samples was conducted using the cetyl trimethylammonium bromide (CTAB) method^[30]^, followed by the sequencing process by BGI Genomics. For library construction, the Illumina TruSeq DNA PCR-Free Library Prep Kit was utilized, with subsequent sequencing carried out using Illumina HiSeq X Ten system.

### Plastome assembly and genome annotation

For the *de novo* assembly of the 22 newly sequenced plastomes, GetOrganelle v.1.6.4 was utilized^[31]^, with a complete plastome of *A. sinensis* (MN720647) serving as the reference. Genome annotation was conducted using CPGAVAS2^[32]^, and the annotation results were subsequently verified manually. The annotation files were uploaded in Figshare (doi:10.6084/m9.figshare.25713024). Finally, the circular maps of the *Aquilaria* plastomes were generated using Chloroplot^[20]^, with visualization facilitated by OGDRAW^[33]^.

### Genome comparison and variation analysis

The analysis of the SC and IR borders among the 22 *Aquilaria* plastomes was conducted using CPJSdraw v1.0^[34]^ to visualize the expansion and contraction of IRs. Gene rearrangement within these plastomes was examined using Mauve v.2.4.0^[35]^. To facilitate comparative analyses, 15 complete *Aquilaria* plastomes were downloaded from GenBank and reannotated (Supplemental Table S1). Subsequently, the mVISTA^[36]^ tool was employed to visualize the similarity among the 37 *Aquilaria* plastomes. Default parameters were utilized for plastome alignment in Shuffle-LAGAN mode. The number of polymorphic sites and nucleotide variability (π) were further evaluated using DnaSP v.6.12^[37]^, employing a step size of 200 bp and a window length of 600 bp.

### Repeat element analysis

REPuter (https://bibiserv.cebitec.uni-bielefeld.de/reputer) online program was employed^[38]^ to detect large sequence repeats (LSRs), including forward (F), reverse (R), complement (C), and palindrome (P) sequence repeats. Parameters were set for sequences of n ≥ 30 bp, a sequence identity of ≥ 90%, and a Hamming distance of 3. Additionally, simple sequence repeats (SSRs) were identified using the MISA-web (https://webblast.ipk-gatersleben.de/misa/) online program^[39]^. Thresholds of seven for mononucleotide repeats, four for dinucleotide repeats, and three for tri-, tetra-, and penta-nucleotide repeats were utilized.

### Evolutionary rates and phylogenetic analysis

In DAMABE v7.3.11^[40]^, 79 protein-coding genes (PCGs) were extracted from 37 plastomes, followed by alignment using MAFFT software (v.7.487)^[41]^. Subsequently, the CodeML in PAML v.4.9^[42]^ was employed to estimate rates of synonymous (Ks) and non-synonymous (Ka) substitution, along with their ratio (Ka/Ks), utilizing a one-ratio model.

Phylogenetic relationships among 37 *Aquilaria* samples were inferred based on complete plastomes with maximum likelihood (ML) and Bayesian inference (BI) methods. To root the trees, three samples from the *Daphne* genus were selected as outgroups. For the ML analysis, RAxML v.8.2.12^[43]^ with the GTRCAT model was employed, performing 1,000 bootstrap replicates to assess node support. Convergence was evaluated using the '-I autoMRE' parameter. For the BI analysis, MrBayes 3.2.7a^[44]^ was utilized. The best models for nucleotide substitutions were selected using ModelTest-NG^[45]^ based on the Bayesian information criterion (BIC). Two trials with four independent Markov chains were run for 10 million generations each, and convergence was verified using Tracer v.1.7.1^[46]^.

## Results

### Plastome genome structure and organization

**Figure 1:**
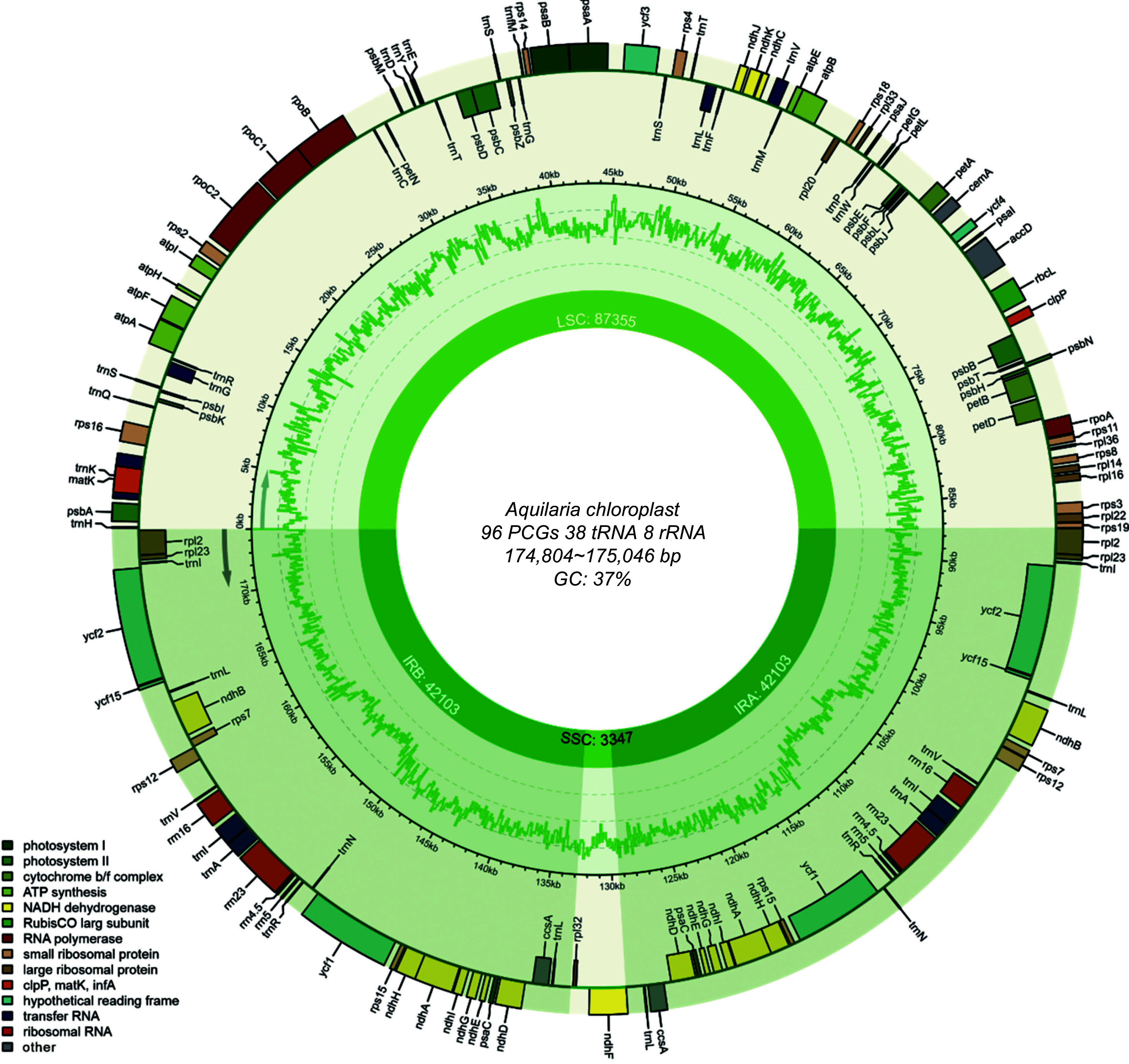
Annotation map of the *Aquilaria* plastome. Genes listed inside and outside the circle are transcribed clockwise and counterclockwise, respectively, with genes color-coded based on functional classification.

In this study, the structural features and plastome organizations of 22 newly sequenced *Aquilaria* plastomes were investigated (Fig. 1, Supplemental Table S1). The length of these plastomes ranged from 174,804 to 175,046 bp, with an overall GC content ranging between 36.70% and 36.71%. Each plastome exhibited a typical quadripartite structure, comprising IRs ranging from 42,095 to 42,172 bp, an SSC region ranging from 3,344 to 3,347 bp and an LSC region ranging from 87,270 to 87,355 bp in size.

The plastomes of these *Aquilaria* samples encoded an identical set of 142 genes (Table 1), comprising 96 PCGs, 38 transfer RNA (tRNA) genes, and eight ribosomal RNA (rRNA) genes. Among them, 64 single-copy PCGs were identified, with 16 PCGs located in the IR regions. Additionally, four rRNA genes (*rrn4.5*, *rrn23*, *rrn5*, and *rrn16*) and eight tRNA genes (*trnA-UGC*, *trnI-CAU*, *trnI-GAU*, *trnL-CAA*, *trnL-UAG*, *trnN-GUU*, *trnR-ACG*, and *trnV-GAC*) were found to have two copies in IR regions. Introns were detected in 10 PCGs and six tRNA genes. Notably, two PCGs, *ycf3*, and trans-spliced *rps12* (characterized by the first exon located in the LSC region and the other two in the IR regions) contained two introns. These findings underscore a highly conserved genome structure and gene content across *Aquilaria* plastomes.

**Table 1:** Genes present within *Aquilaria* plastomes.

Category	Group of genes	Names of genes
Photosynthesis	Subunits of photosystem I	*psaA*, *psaB*, *psaC*(2), *psaI*, *psaJ*
	Subunits of photosystem II	*psbA*, *psbB*, *psbC*, *psbD*, *psbE*, *psbF*, *psbH*, *psbI*, *psbJ*, *psbK*, *psbL*, *psbM*, *psbN*, *psbT*, *psbZ*
	Subunits of NADH dehydrogenase	ndhA*(2), ndhB*(2), *ndhC*, *ndhD*(2), *ndhE*(2), *ndhF*, *ndhG*(2), *ndhH*(2), *ndhI*(2), *ndhJ*, *ndhK*
	Subunits of cytochrome b/f complex	petA, petB*, petD*, petG, petL, petN
	Subunits of ATP synthase	atpA, atpB, atpE, atpF*, atpH, atpI
	Large subunit of rubisco	*rbcL*
	Subunits photochlorophyllide reductase	−
Self-replication	Proteins of large ribosomal subunit	*rpl14*, *rpl16*, rpl2*(2), *rpl20*, *rpl22*, *rpl23*(2), *rpl32*, *rpl33*, *rpl36*
	Proteins of small ribosomal subunit	*rps11*, rps12**(2), *rps14*, *rps15*(2), rps16*, *rps18*, *rps19*, *rps2*, *rps3*, *rps4*, *rps7*(2), *rps8*
	Subunits of RNA polymerase	*rpoA*, *rpoB*, rpoC1*, *rpoC2*
	Ribosomal RNAs	*rrn16*(2), *rrn23*(2), *rrn4.5*(2), *rrn5*(2)
	Transfer RNAs	trnA-UGC*(2), *trnC-GCA*, *trnD-GUC*, *trnE-UUC*, *trnF-GAA*, *trnG-GCC*, trnG-UCC*, *trnH-GUG*, *trnI-CAU*(2), trnI-GAU*(2), trnK-UUU*, *trnL-CAA*(2), trnL-UAA*, *trnL-UAG*(2), *trnM-CAU*, *trnN-GUU*(2), *trnP-UGG*, *trnQ-UUG*, *trnR-ACG*(2), *trnR-UCU*, *trnS-GCU*, *trnS-GGA*, *trnS-UGA*, *trnT-GGU*, *trnT-UGU*, *trnV-GAC*(2), trnV-UAC*, *trnW-CCA*, *trnY-GUA*, *trnfM-CAU*
Other genes	Maturase	*matK*
	Protease	*clpP*
	Envelope membrane protein	*cemA*
	Acetyl-CoA carboxylase	*accD*
	c-type cytochrome synthesis gene	*ccsA*
	Translation initiation factor	−
	other	−
Genes of unknown function	Conserved hypothetical chloroplast ORF	*ycf1*(2), *ycf15*(2), *ycf2*(2), ycf3**, *ycf4*
Gene*: Gene with one intron; Gene**: Gene with two introns; Gene(2): Number of copies of multi-copy genes.		

### Comparative genomics analysis and identification of highly variable regions

**Figure 2:**
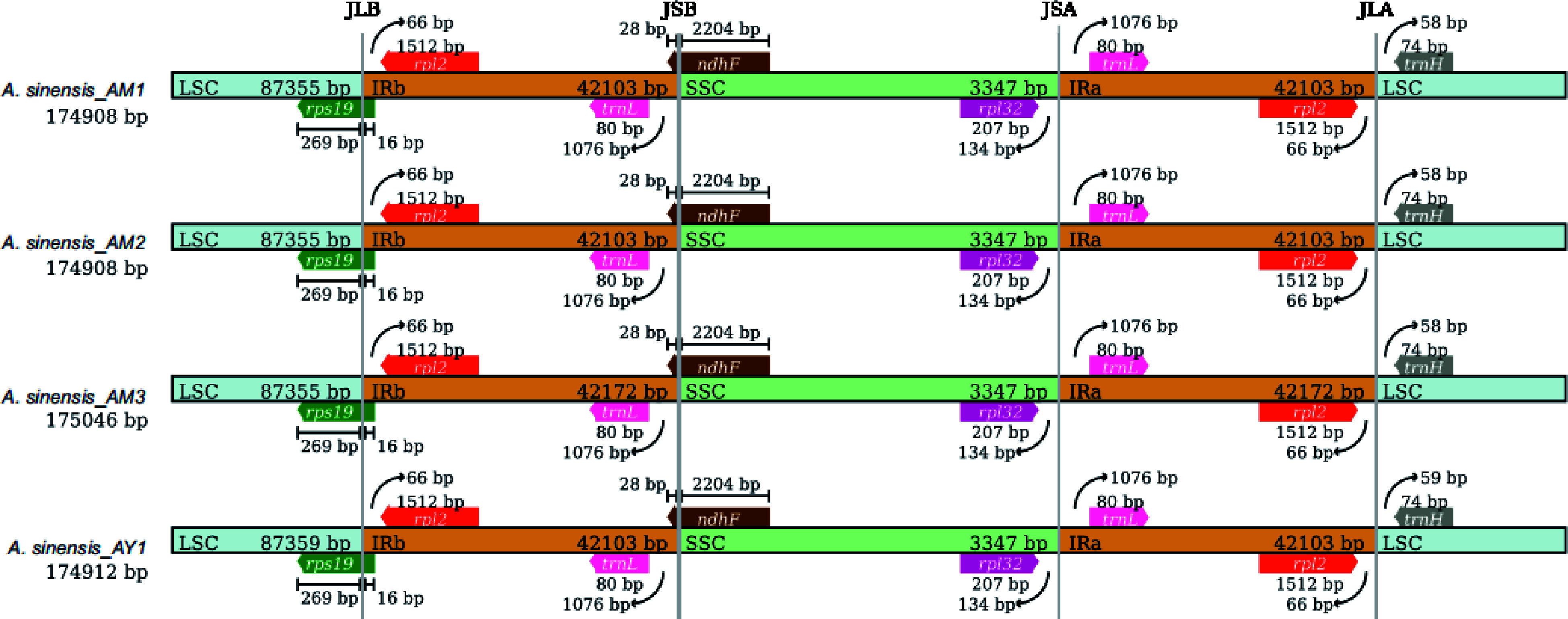
Comparison of the large single-copy (LSC), small single-copy (SSC), and inverted repeat (IR) region borders of *Aquilaria* plastomes. The LSC, IR, and SSC regions are depicted with blue, orange, and green blocks, respectively. Gene boxes above the block are transcribed counterclockwise while those below the block are transcribed clockwise. (Representative plastomes from Supplemental Fig. S2).

The Mauve alignment results (Supplemental Fig. S1) revealed no gene rearrangements in these newly sequenced plastomes, with the plastome of *A. malaccensis* serving as the reference. To further explore structure variations within the *Aquilaria* genus, 15 *Aquilaria* plastomes were downloaded from GenBank for comparative analysis. The junction sites on the boundaries of genomic regions in *Aquilaria* species, namely LSC/IRb, SSC/IRb, SSC/IRa, and LSC/IRa, are pivotal in chloroplast genome evolution. These junctions were determined using CPJSdraw. As depicted in Fig. 2 (or Supplemental Fig. S2), the boundaries of genomic regions in *Aquilaria* samples are highly conserved, with only minor variations observed in the junctions and adjacent genes. The *rps19* gene was found across both the LSC and IRb regions, with most of its sequence situated in the LSC region. Furthermore, a 16 bp extension of this gene was consistently observed in the IRb region across all *Aquilaria* plastomes. This 16 bp extension could potentially serve as a genus-special marker for *Aquilaria*. Moreover, it was observed that the IRb/SSC junction was located within the *ndhF* gene. Notably, no genes were found spanning the SSC/IRa and IRa/LSC junctions.

The overall organization of the plastomes was analyzed using mVISTA, revealing a high degree of conservation among these *Aquilaria* plastomes (Supplemental Fig. S3). Interestingly, the SC regions exhibited higher divergence compared to the IR regions. Furthermore, it was observed that PCGs displayed a lower level of variation when compared to non-coding regions.

**Figure 3:**
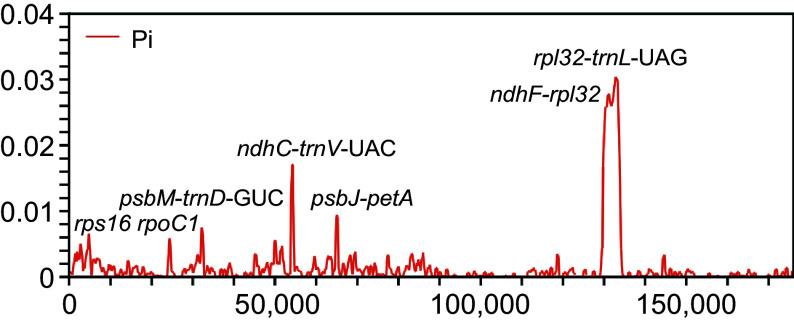
Comparison of nucleotide variability values among 37 plastomes using window sliding analysis. The x-axis indicates the position of the midpoint of the window, while the y-axis indicates the nucleotide diversity of each window.

Furthermore, we identified some variant hotspot regions, primarily comprising intergenic regions and a few gene coding regions such as *rps16* and *rpoC1*. The nucleotide variability (π) values across the analyzed coding and intergenic sequences of the 37 chloroplast genomes ranged from 0 to 0.03037 (Fig. 3). Notably, variation in the SSC region was higher than that in the IR region, consistent with the mVISTA results. Seven regions with π values higher than 0.005, including *rps16*, *rpoC1*, *psbM*-*trnD*-GUC, *ndhC*-*trnV*-UAC, *psbJ*-*petA*, *ndhF*-*rpl32*, and *rpl32*-*trnL*-UAG, were detected. These regions were identified as HVRs in this study. Among them, the coding region of the *rps16* gene exhibited a π value exceeding 0.04, while the *rpl32*-*trnL*-UAG inter-region possessed the most informative sites. These HVRs have the potential to serve as barcodes for identifying *Aquilaria* species.

### Repeat elements analysis

**Figure 4:**
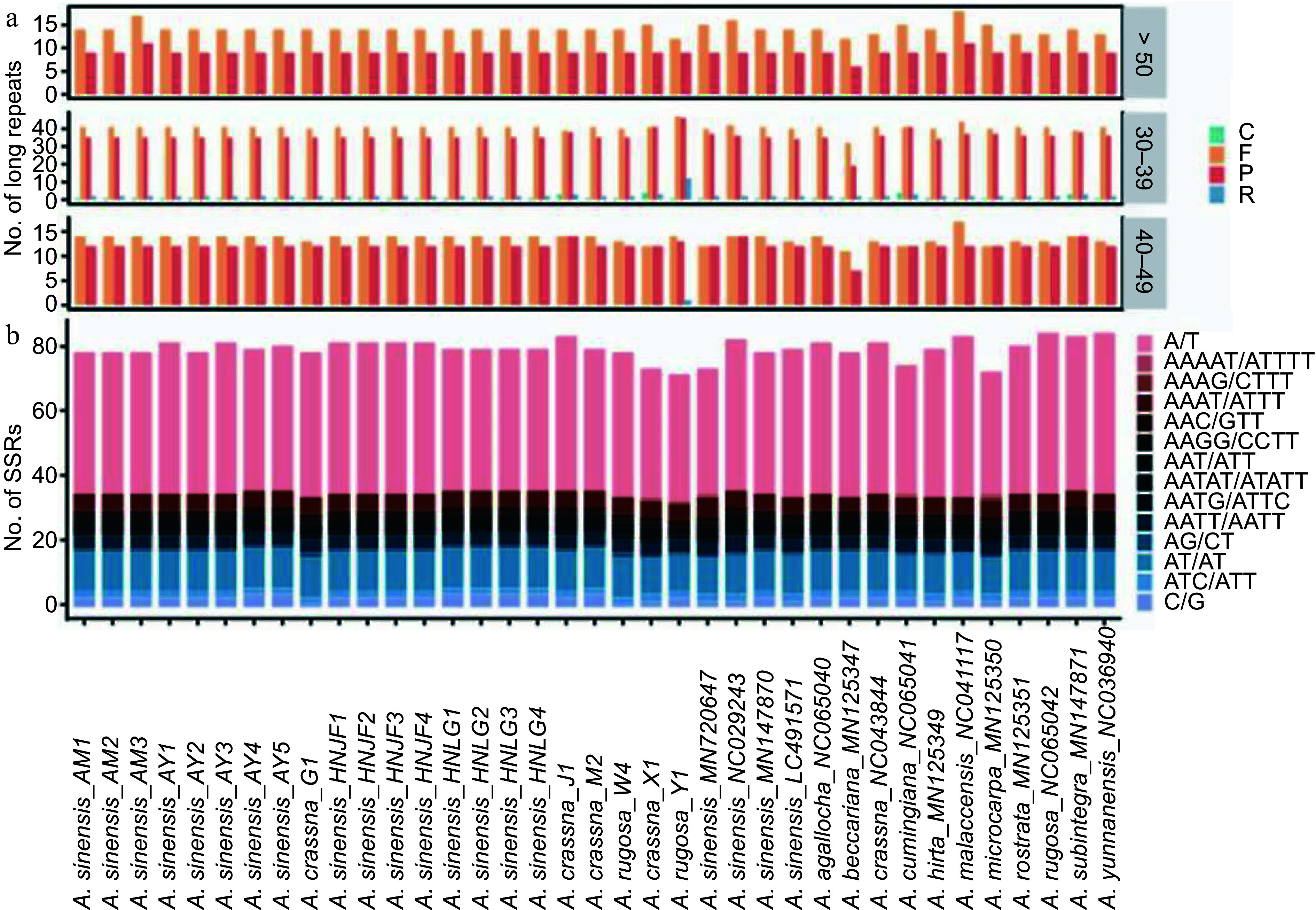
(a) Distribution of different types of large sequences repeat (LSRs) in *Aquilaria* plastomes, including complementary (C), forward (F), palindromic (P), and reverse (R) type repeats. (b) Distribution of distinct types of simple sequence repeats (SSRs) in *Aquilaria* plastomes.

Nucleotide repeats within chloroplast genomes play a crucial role in plant typing and are widely used as genetic molecular markers in population genetics. Large sequence repeats (LSRs) were identified as repeats with a length of ≥ 30 bp each. A total of 4,765 LSRs were detected across the 37 plastomes, encompassing palindromic repeats (P), forward repeats (F), reverse repeats (R) and complementary repeats (C) (Fig. 4a). Among these, forward repeats were the most prevalent type, with 2,533 occurrences, while complementary repeats were the least common, totaling 46 occurrences.

In the MISA-web analysis, five types of simple sequence repeats (SSRs) were identified, with mononucleotide repeats comprising the majority (1,775, 59.91%), followed by dinucleotide repeats (477, 16.1%), trinucleotide repeats (294, 9.92%), tetranucleotide repeats (409, 13.80%), and pentanucleotide repeats (8, 0.27%) (Fig. 4b). Among these SSRs, repeat units of A/T, AT/AT, AAAT/ATTT, AAAAT/ATTTT, and AAATT/AATTT accounted for 87.55% of the total, indicating a bias towards A/T bases in SSR composition.

### Selection pressure and phylogenetic analysis

**Figure 5:**
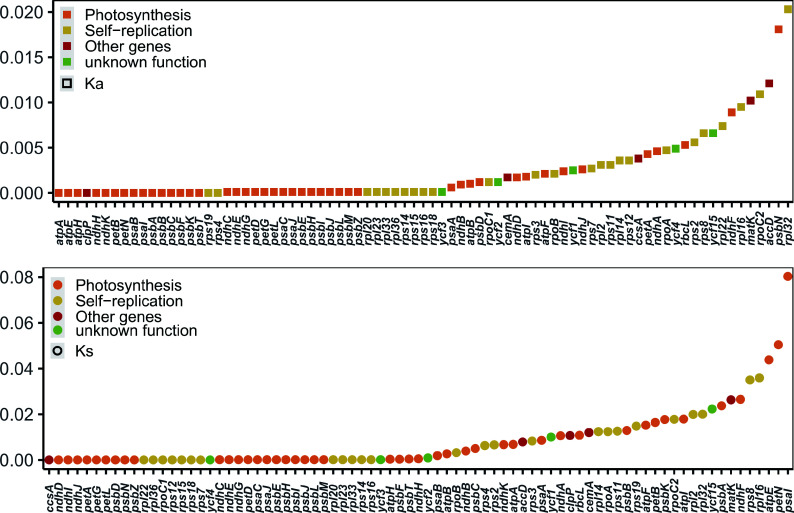
Values of synonymous (Ks) and non-synonymous (Ka) substitution rates for 79 protein-coding genes in *Aquilaria*.

Ka/Ks is a valuable metric for evaluating whether PCGs have undergone adaptive evolution. Typically, synonymous nucleotide substitutions occur more frequently than non-synonymous substitutions in most genes of organisms, resulting in Ka/Ks values typically being less than 1. In this study, the Ka/Ks values for the PCGs of these plastomes were calculated. Most of the PCGs exhibited Ka/Ks values of less than 1, indicating that these species were subjected to strong purifying selection during the long-term evolutionary process (Fig. 5). Notably, the expression-related *accD* gene displayed the highest Ka/Ks value (1.54). This finding suggests that the *accD* gene may have been influenced by positive selection during the evolutionary process.

**Figure 6:**
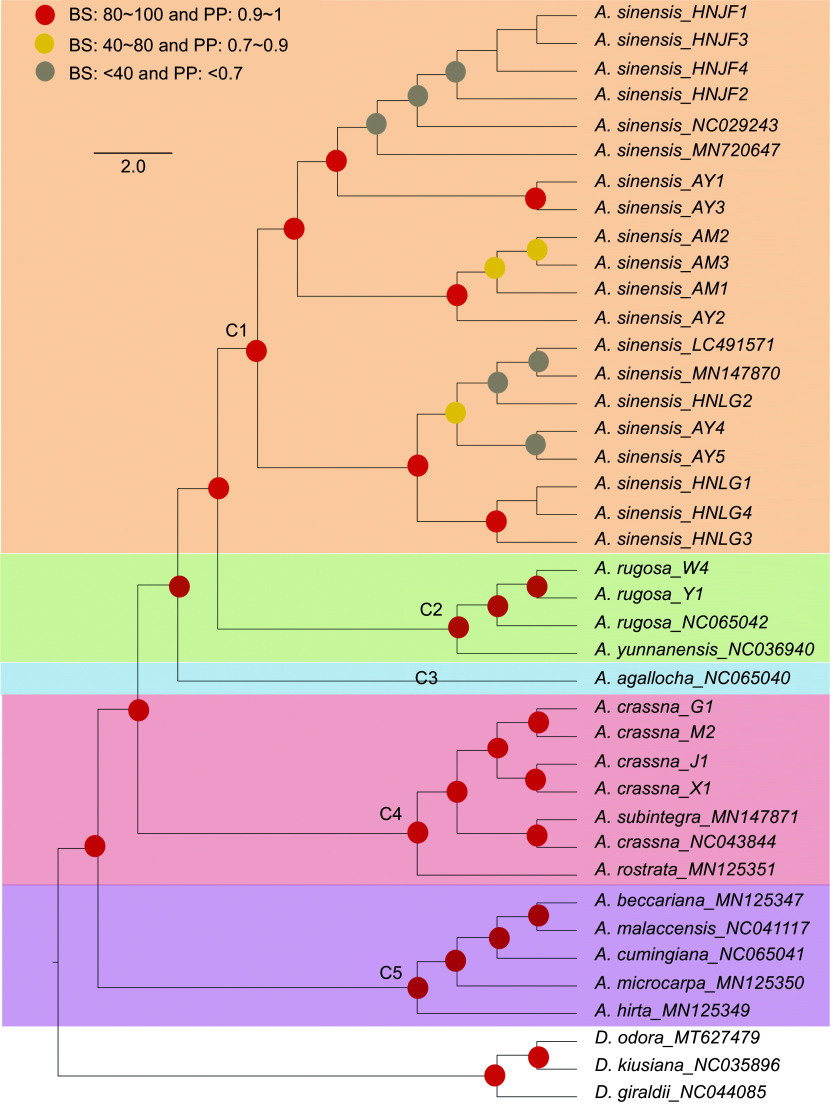
Phylogenetic tree depicting relationships among *Aquilaria* species. Red circles indicates bootstrap support (BS) values greater than 80% and posterior probability (PP) values exceeding 1. Yellow circles indicates BS values ranging from 40% to 80%, and PP values ranging from 0.7 to 0.9. Grey circles indicates BS values below 40% and PP values less than 0.7.

To elucidate the phylogenetic relationships within the *Aquilaria* genus, 22 newly sequenced *Aquilaria* plastomes were utilized in conjunction with an additional 15 *Aquilaria* plastomes to reconstruct the phylogenetic tree using ML and BI methods. Additionally, three *Daphne* samples were selected as outgroups (Fig. 6). The results revealed the formation of five clades within *Aquilaria*. The newly sequenced *A. sinensis* plastomes clustered together with published *A. sinensis* plastomes, forming a monophyletic clade designated as clade 1 (C1). Clade 2 (C2) comprised three *A. rugosa* plastomes, which clustered together and formed a sister group to *A. yunnanensis* with high confidence. Clade 3 (C3) consisted of only one species, identified as *A. agallocha.*

In clade 4 (C4), it was observed that *A. crassna* (NC043844) clustered together with *A. subintegra* (MN147871), which aligns with previous research^[47]^. The newly sequenced *A. crassna* plastomes also clustered together, demonstrating high consistency. This finding suggests the possibility of hybridization events or misidentification of the published plastome sample (NC043844). It is conceivable that hybridization occurred among these species, with plastomes likely following maternal inheritance, leading to significant disturbance in DNA information within the plastomes. Alternatively, the high similarity in morphology between these species may have contributed to misidentification. Lastly, clade 5 (C5) encompassed five species, mirroring the phylogenetic findings of a previous study^[29,47]^.

## Discussion

In this study, the complete plastome sequences of 22 *Aquilaria* taxa were presented. By integrating these newly sequenced plastomes with an additional 15 *Aquilaria* published plastomes, a series of comparative analyses within this genus were conducted. The investigation into the location of the SC/IR boundaries offers novel insights into evolutionary studies. The expansion and contraction of IR regions are often associated with various evolutionary events, including gene duplication, loss of gene copies, and the emergence of pseudogenes (such as the *ycf1* pseudogene observed in many plants)^[48,49]^. The substantial variation observed among these boundaries may harbor potential markers for genus/species identification^[50,51]^. The present research has unveiled a consistent structural feature at the IR/SC boundary in *Aquilaria* species. Intriguingly, all *Aquilaria* plastomes analyzed in this study exhibited a 16 bp extension of the *rps19* gene in the IRb region. This phenomenon is rarely observed in other species within the Thymelaeaceae family, as it has only been detected in a few species across certain genera^[52,53]^. In *Aquilaria*, a consistent 16 bp extension was observed across all examined species, suggesting its potential as a genus-specific marker. Such markers have been utilized for identification previously; for example, the 110 bp expansion of the *rps19* gene in the Crassulaceae family serves as a specific family marker^[19]^. Future studies employing extensive datasets of Thymelaeaceae species may identify additional family-specific identification markers. Furthermore, discrepancies were noted in the plastomes of *A. malaccensis* (NC041117) and other *Aquilaria* species, particularly in the positions and transcriptional directions of *ndhF* and *rpl32*; their transcribed directions were reversed. These anomalies were primarily attributed to assembly errors. Notably, the same misassembly was found in *A. sinensis* (NC029243). The loss of *rps32* and the incorrect positioning of *trnN*, *ndhF*, and *ycf1* genes further underscored the erroneous assembly of the plastome. To elucidate the authentic structural patterns of the plastome genome, these inaccurate annotations were substituted with normal data, thereby eliminating potential sources of misinterpretation.

Furthermore, *accD* displayed the highest Ka/Ks value of 1.54. Previous research has suggested that adaptive evolution may occur at the molecular level, as evidenced by an elevated Ka/Ks value^[54]^. Numerous studies have corroborated that a higher Ka/Ks ratio correlates with a more robust positive selection^[26,55,56]^. Thus, the elevated Ka/Ks ratio observed in *accD* implies its significant contribution in the adaptation and evolutionary processes within *Aquilaria* species.

While previous research has attempted to elucidate the phylogenetic status of the *Aquilaria* genus, the limited availability of plastomic resources for *Aquilaria* has left certain issues unresolved. One such point of interest pertains to the relationship among *A. sinensis*, *A. agallocha*, and *A. yunnanensis*. Farah et al.^[28]^ utilized two types of datasets (chloroplast loci and ITS regions) and reported conflicting patterns among these three species. The chloroplast loci data clustered these species into the same clade, with *A. agallocha* being closer to *A. sinensis*. Conversely, the evolutionary tree based on ITS regions separated *A. sinensis* to another clade. Another study^[29]^ also examined these species for phylogenetic analysis, yielding different results. In that study, these species were clustered in the same clade, with *A. yunnanensis* exhibiting a closer relationship to *A. sinensis*. Additionally, several studies^[1,47,57]^ have explored phylogenetic relationships in *Aquilaria*; however, not all of them included all three species of interest. The phylogenetic tree reconstructed in this study mirrored the results of the study conducted by Lee et al.^[29]^ with high confidence. The structure of the phylogenetic tree appeared to be highly influenced by the volume of the dataset; as the number of *Aquilaria* plastomes were increased, it was anticipated that this result would better elucidate the true relationship among these species.

Furthermore, of interest was a previous study^[47]^ which revealed that different varieties of *A. crassna* did not form a monophyletic clade, as *A. subintetegra* (MN147870) clustered with *A. crassna* (MK779998, also coded as NC043844). To explore the underlying reason for this discrepancy, four *A. crassna* plastomes were sequenced. The phylogenetic results unveiled that the newly sequenced *A. crassna* plastomes in this study clustered together. However, the published *A. crassna* (NC043844) also clustered with *A. subintegra* (MN147871), aligning with the findings of the earlier research conducted by Hishamuddin et al.^[47]^. Chloroplasts, organelles present in plants and some protist cells, possess their own DNA distinct from the nuclear genome. The similarity of the chloroplast genome sequences can unveil phylogenetic relationships and evolutionary history among species^[58−62]^, suggesting inheritance from a common ancestor in many cases^[63−65]^, as chloroplasts are typically maternally inherited in plants^[66,67]^. Conversely, similar plastomes may also result from convergent evolution, where analogous chloroplast genome sequences evolve independently on distinct evolutionary trajectories. In the case of *A. crassna* (NC043844), there is a possibility of incorrect assembly, or even misidentification of the specimen as either *A. crassna* (NC043844) or *A. subintegra* (MN147871). Moreover, conducting a pan-plastome study involving *A. subintegra* and *A. crassna* could offer a more comprehensive understanding of the relationship between these two species.

With the augmented dataset of plastomes in this study, a more distinct phylogenetic pattern was unveiled compared to previous research. Besides, although plastomes can provide valuable insights into phylogenetic relationships, the limitations imposed by frequent artificial hybridization are recognized^[8]^. It is anticipated that future studies integrating data from nuclear genomes, mitogenomes, and plastomes will undoubtedly provide a deeper understanding of the phylogenetic implications within the *Aquilaria* genus. This study can serve as a valuable example for species identification in trees, highlighting the significance of understanding evolutionary relationships between species or subspecies, given the high similarity among tree species.

## Conclusions

This study presents the sequencing of 22 *Aquilaria* plastomes, revealing no gene rearrangements among them. Comparative genomics unveiled a consistent genome size (174,804–175,046 bp) and GC content (36.70%–36.75%) across *Aquilaria* plastomes. Additionally, seven HVRs were identified, spanning *rps16*, *rpoC1*, *psbM*-*trnD*-GUC, *ndhC*-*trnV*-UAC, *psbJ*-*petA*, *ndhF*-*rpl32*, and *rpl32*-*trnL*-UAG. In examining the IR junction pattern, it was noted that the *rps19* gene spans both the LSC and IRb regions, with a 16 bp expansion of the *rps19* gene in IRb, potentially serving as a genus-specific marker for *Aquilaria*. Furthermore, the present analysis identified four types of LSRs and five types of SSRs with a notable A/T bias. The *ndhD* gene displayed the highest Ka/Ks value of 1.54, indicative of its significant contribution to the adaptation and evolution of *Aquilaria* species. Finally, the present study elucidated the phylogenetic relationships within *Aquilaria* and addressed several issues within the genus through an expanded dataset, providing valuable insights into its evolutionary dynamics.

## SUPPLEMENTARY DATA

Supplementary data to this article can be found online.

## Data Availability

The annotation files of newly assembled *Aquilaria* plastomes are uploaded to the Figshare website (https://figshare.com/, doi:10.6084/m9.figshare.25713024).
