# Supplementary material for: Insights into Aquilaria phylogenetics through comparative plastomic resources
Source: For Res (Fayettev). 2024 Sep 4;4:e030. doi: 10.48130/forres-0024-0028 (PMC11524301; doi:10.48130/forres-0024-0028)
Supplement: Supplementary file 1 — Supplementary data to this article can be found online. [file forres-0024-0028-S1.zip › 10.48130_forres-0024-0028-Suppl-FigureS2.pdf]

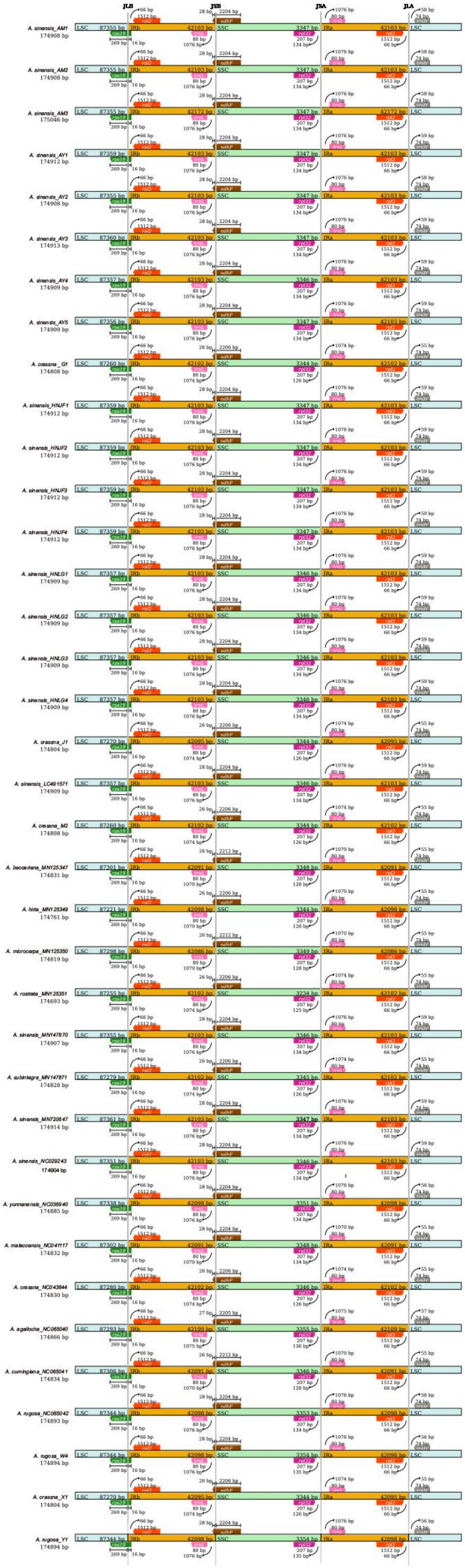

Fig S2. Comparison of the large single-copy (LSC), small single-copy (SSC), and inverted repeat (IR) region borders of *Aquilaria* plastomes. The LSC, IR, and SSC regions are depicted with blue, orange, and green blocks, respectively. Gene boxes above the block are transcribed counterclockwise while those below the block are transcribed clockwise.
