# Supplementary material for: Insights into Aquilaria phylogenetics through comparative plastomic resources
Source: For Res (Fayettev). 2024 Sep 4;4:e030. doi: 10.48130/forres-0024-0028 (PMC11524301; doi:10.48130/forres-0024-0028)
Supplement: Supplementary file 1 — Supplementary data to this article can be found online. [file forres-0024-0028-S1.zip › 10.48130_forres-0024-0028-Suppl-FigureS3.pdf]

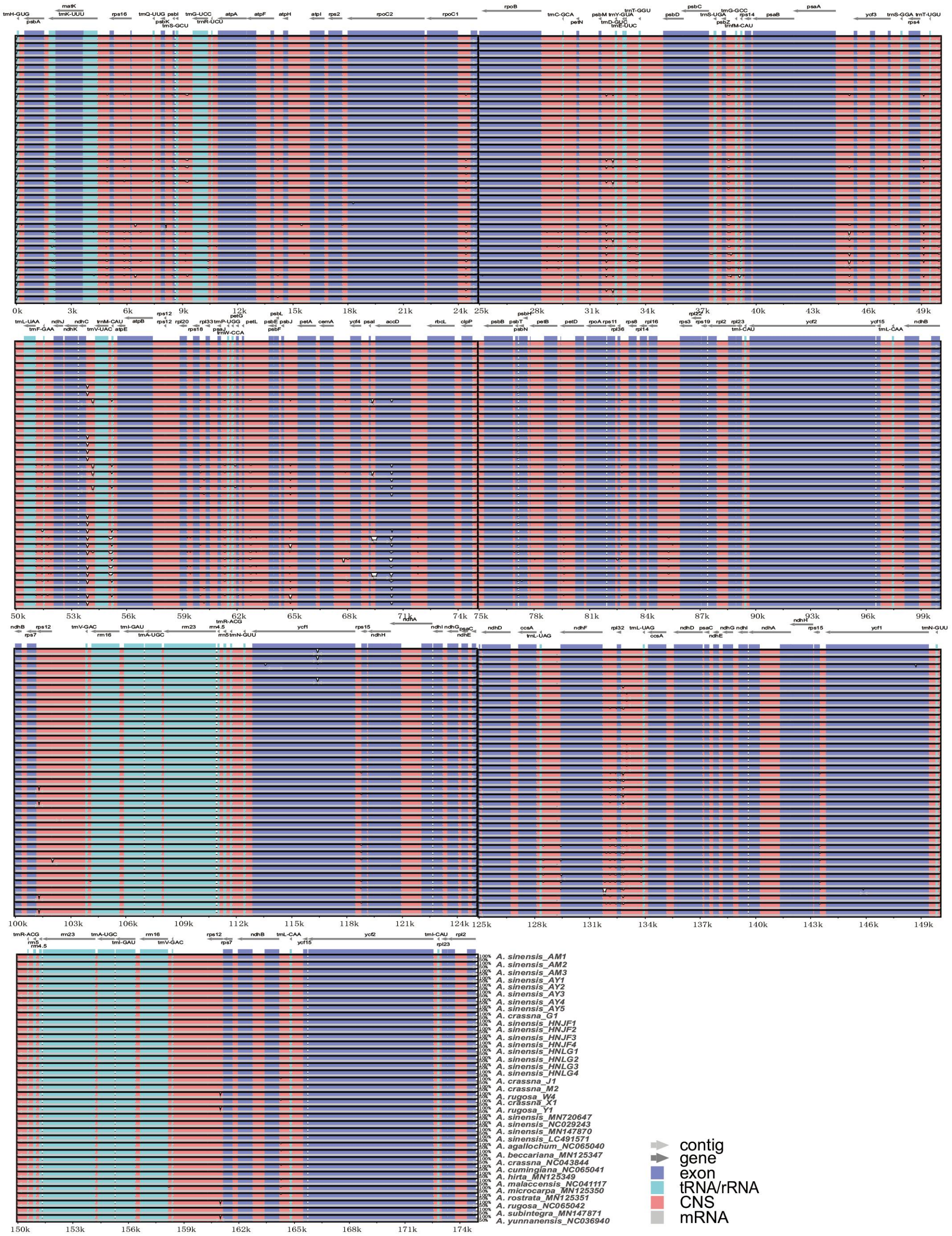

Fig S3. Structural comparisons of 37 *Aquilaria* plastomes with the mVISTA program. X-axis represents the gene position and the y-axis means the con-sistency of the sequences with the numbers ranged from 50% to 100%.
