# Supplementary material for: Insights into Aquilaria phylogenetics through comparative plastomic resources
Source: For Res (Fayettev). 2024 Sep 4;4:e030. doi: 10.48130/forres-0024-0028 (PMC11524301; doi:10.48130/forres-0024-0028)
Supplement: Supplementary file 1 — Supplementary data to this article can be found online. [file forres-0024-0028-S1.zip › 10.48130_forres-0024-0028-Suppl-FigureS1.pdf]

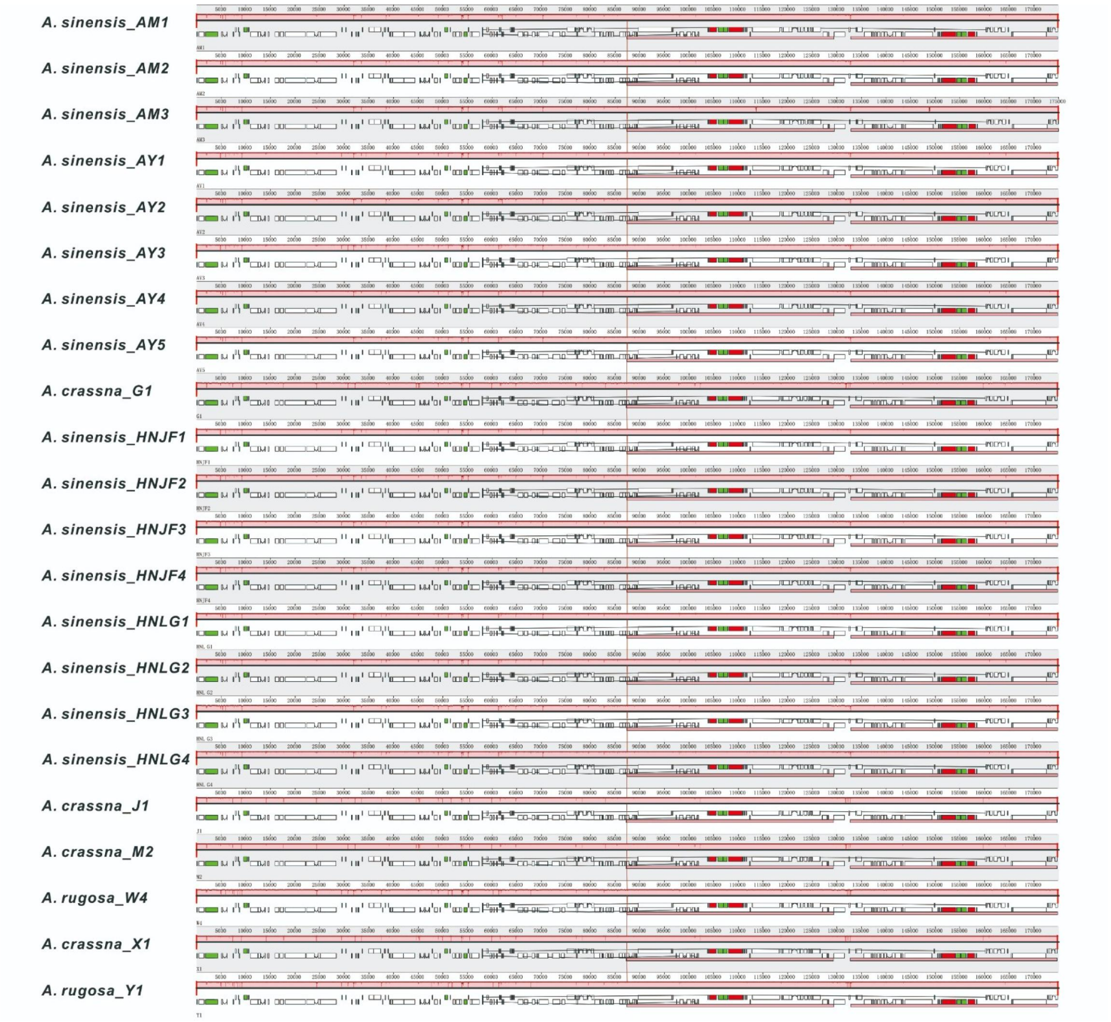

Fig S1. Mauve alignment of the 22 *Aquilaria* samples alliance plastomes, illustrating colliner blocks rearrangement of the accessible gene at the junction of inverted repeat and small-single copy regions. White blocks represent protein-coding gene, black blocks represent transfer RNA (tRNA) genes, and red blocks represent ribosomal RNA (rRNA).
